# Hepatic Sarcoidosis Complicated with Pancreatic Adenocarcinoma

**DOI:** 10.1155/2019/9383019

**Published:** 2019-11-19

**Authors:** Kamesh Gupta, Tuyyab Hassan, Shahid Rizwan, Bandhul Hans, Rahul Jawale, David Desilets

**Affiliations:** ^1^Department of Internal Medicine, UMMS-Baystate, Springfield, MA, USA; ^2^Department of Gastroenterology, UMMS-Baystate, Springfield, MA, USA; ^3^Depeartment of Internal Medicine, Walia Hospital, Ludhiana, India; ^4^Department of Pathology, UMMS-Baystate, Springfield, MA, USA

## Abstract

Sarcoidosis is a systemic noncaseous granulomatous disease. The liver is a common location but usually asymptomatic. Current literature suggests an association between sarcoidosis and cancers. However, there is a lack of definite evidence. We present a case of a 59-year-old man with jaundice and acutely elevated alkaline phosphatase. The diagnosis was confirmed by obtaining a liver biopsy and was treated with 6 months of steroids. A year later, he had a recurrence of jaundice. MRCP showed biliary dilatation and a mass in the pancreatic head, confirmed by biopsy to be adenocarcinoma. This is the first case to be reported of hepatic sarcoidosis associated with pancreatic cancer.

## 1. Introduction

Sarcoidosis is a multisystem disease marked by persistent noncaseating granulomas in the organs. The most common sites affected are the intrathoracic lymph nodes and lungs. However, hepatic involvement is also common and is observed in 50–80% of patients with systemic sarcoidosis. Sarcoidosis appears to be associated with a significantly increased risk for cancer in affected organs.

## 2. Case Presentation

**Figure 1:**
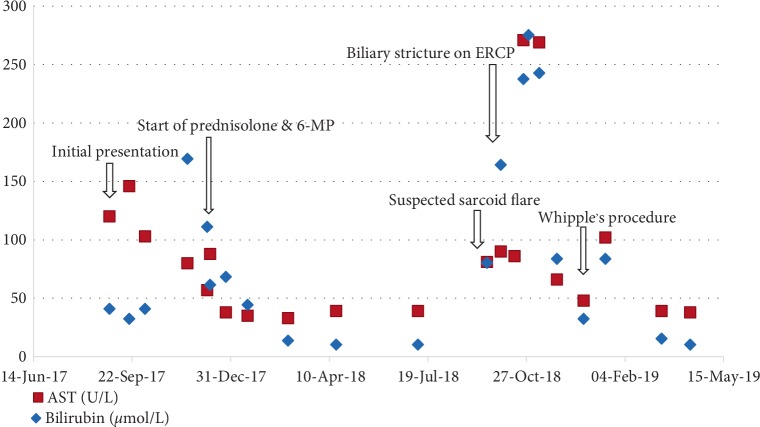
Bilirubin and ALT trend in relation to patient's diagnosis and treatment.

Our patient was a relatively healthy 59-year-old African-American male who presented with several weeks of pruritus, jaundice, nausea, and weight loss. His past medical history was significant for hypertension and he did not consume alcohol, IV drugs, or herbal supplements. Physical findings included jaundice, without signs of chronic liver disease, and a benign abdomen without hepatosplenomegaly or ascites. Labs showed elevated liver enzymes in an obstructive pattern (Figure 1).

**Figure 2:**
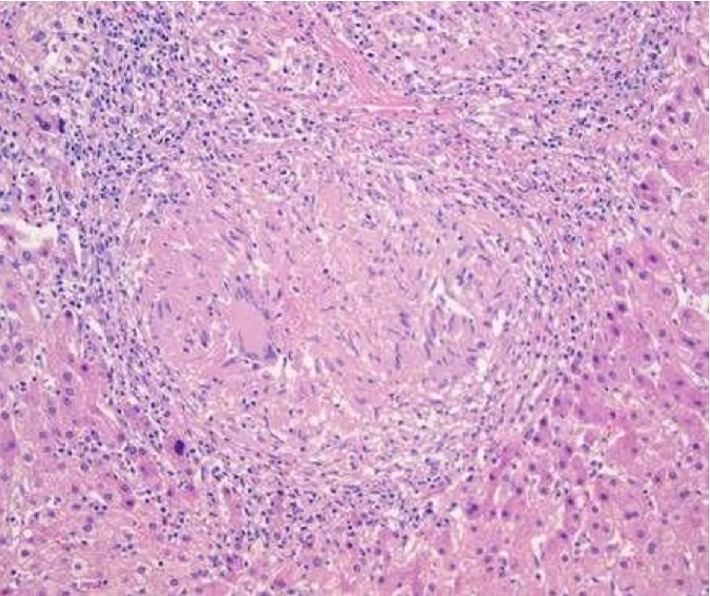
The biopsy demonstrates mildly distorted hepatic architecture due to marked inflammation and presence of granulomas. There is cholestasis. Many non-necrotizing epithelioid granulomas, some confluent, are seen in the portal tracts, periportally and in the lobules. Portal tracts demonstrate mild acute and chronic inflammation (in association with granulomas), bile duct damage and mild proliferation of bile ductules accompanied by neutrophils. Ceroid-laden macrophages are seen in the lobules. There is periportal fibrous expansion and focal portal-portal bridging.

He underwent extensive testing for infectious causes which was negative, including hepatitis A, B, C, E, leptospirosis, VDRL, and peripheral smear performed for malaria. Further evaluation including ANA, Smooth muscle antibody, Antimitochondrial antibodies, IgG elevated antibodies, alpha-1 antitrypsin, and carbohydrate antigen 19-9 were all negative. Ultrasound showed only hepatic steatosis, though further abdominal CT showed an enlarged celiac axis lymph node (>1 cm) and portal lymphadenopathy. There was no duct dilatation or either intrahepatic or extrahepatic ducts on endoscopic ultrasound. Cytology from the enlarged celiac node showed small lymphocytes, not diagnostic for lymphoma. An ultrasound-guided liver biopsy was therefore performed (Figure 2).

**Figure 3:**
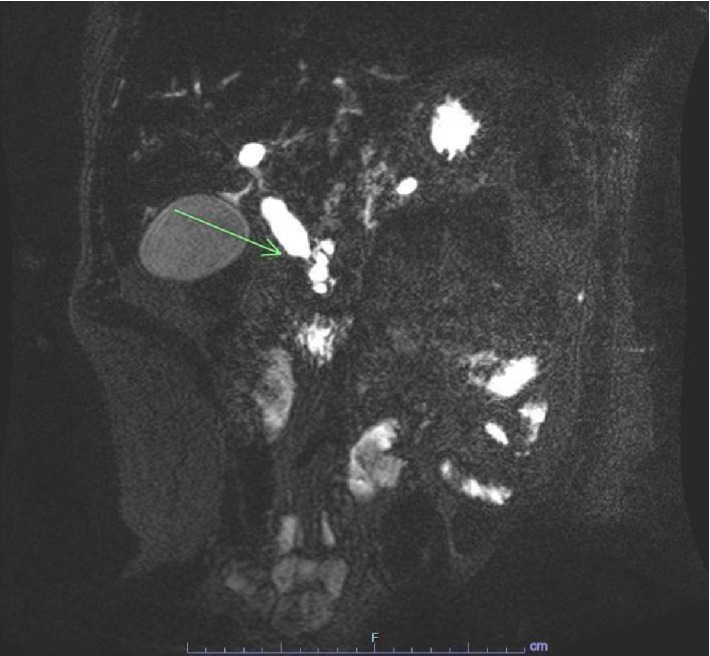
MRI abdomen-enhancing mass in the region of the ampulla, with resulting significant dilation of the biliary tree and the main pancreatic duct.

This showed noncaseating granulomas and bile duct damage, highly suggestive of sarcoidosis. Sarcoidosis was further confirmed with high activity of angiotensin-converting enzyme and mediastinal/hilar lymphadenopathy in CT of the chest. The patient was started on budesonide and 6-mercaptopurine therapy with marked improvement of symptoms. Repeat labs showed resolution of ACE inhibitor levels and liver chemistries except ALP (Figure 1). He underwent spirometry which showed a mild reduction in FEV1/FVC (66%). After approximately 8 months of being asymptomatic on treatment, he was seen in the emergency room for complaints of yellowing of skin, itching, pale stools, and weight loss, reportedly going on for the past 6 months. Laboratory findings showed worsening hepatic function with severe direct hyperbilirubinemia. He was started on methylprednisolone for a suspected sarcoidosis flare-up, with no improvement. He underwent an abdominal ultrasound which showed a dilated common bile duct measuring 1.6 cm and biliary sludge, which was not present before. Magnetic Resonance Cholangiopancreatography revealed persistently enlarged perihepatic and periportal lymph nodes and a mass in the ampulla of the pancreas (Figure 3).

**Figure 4:**
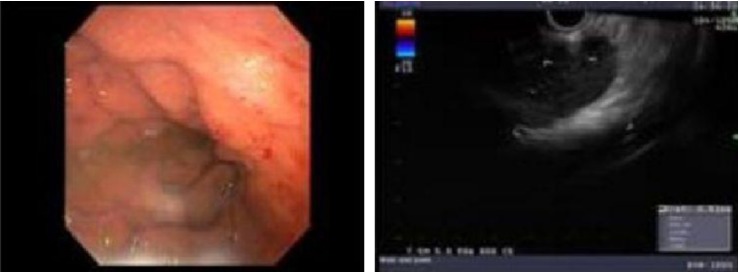
Upper endoscopy with EUS guided biopsy: A large celiac node, measuring about 4 cm in diameter, along with multiple enlarged lymph nodes noted in the porta hepatis and the lesser sac.

**Figure 5:**
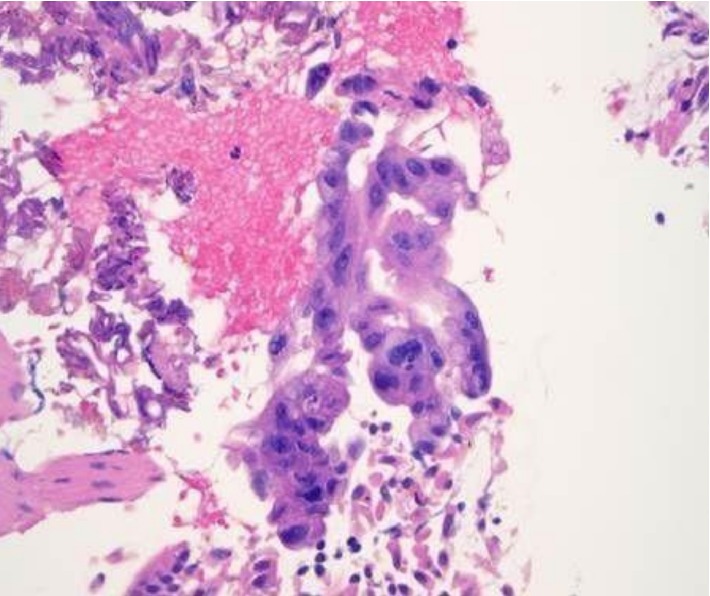
Biliary stricture adenocarcinoma at 400X. Small cluster of atypical glandular cells present, which is highly suspicious for adenocarcinoma. The cytopathology report from brushings showed adenocarcinoma.

Endoscopic Retrograde Cholangiopancreatography showed a biliary stricture and a double-duct sign (proximal pancreatic and common bile duct dilation) with biliary stricture biopsy displaying a cluster of atypical glandular cells, likely adenocarcinoma (Figures 4 and 5). Workup for malignancy included CT PET showing multiple hepatic lesions, the largest measuring 1.3 cm along with other known lymphadenopathy and pancreatic mass. Staging diagnostic laparoscopy showed no evidence of carcinomatosis during which a wedge liver biopsy was taken which showed granulomatous hepatitis, but keratin cocktail immunohistochemistry was negative for carcinoma. He underwent a pancreaticoduodenectomy and antrectomy with no complications.

The pancreatic mass biopsy confirmed pancreatobiliary origin of carcinoma. Three out of 28 lymph nodes were positive for malignant cells and showed evidence of sarcoidosis. The final staging was pT3b pN1, stage 3A. Immunohistochemistry studies revealed a mismatch repair proteins expressed, MSS. He was started on adjuvant chemotherapy and pancreatic enzyme replacement.

## 3. Discussion

The prevalence of hepatic sarcoidosis is often underestimated. From reports, approximately 35–40% of all sarcoidosis patients have abnormal liver function tests, most commonly elevated alkaline phosphatase levels, and 13% have solitary hepatic disease [1, 2]. Patients can present with abdominal pain, jaundice, nausea, vomiting, and hepatosplenomegaly, but most patients are asymptomatic [3]. In severe cases, hepatic sarcoidosis can lead to cholestasis, portal hypertension, or cirrhosis [4]. Liver biopsy is recommended for patients suspected of having symptomatic hepatic sarcoidosis (e.g., elevated serum aminotransferase and alkaline phosphatase) [5]. Other diseases that can cause hepatic granulomas, such as fungal infections, tuberculosis, primary biliary cirrhosis, lymphoma, and drug reactions, should be excluded. Another important differential is IgG4 related disease (IgG4 RD) which can be differentiated radiologically based on absence of a diffusely enlarged “sausage-shaped” pancreas, and a halo of edema surrounding the organ and delayed homogenous enhancement during the portal and venous phases [6, 7]. However, biopsy remains the gold standard to differentiate IgG4 RD and hepatic sarcoidosis. But in cases where a biopsy is potentially difficulty, a short trial of prednisolone is a prudent approach. Other tests that can aid diagnosis include elevation of angiotensin-converting enzyme (ACE levels) and hypercalcemia [8]. Radiological studies, such as ultrasonography or Computed Tomography, may show hepatomegaly or hypoattenuated nodules in the liver. These nodules may be confused with liver metastasis or other granulomatous diseases [9]. Extrinsic compression of the biliary tree from mass effect of sarcoid granulomas has also been reported [10]. Sarcoidosis appears to occur with various hematologic malignancies and solid tumors [11], for example, the relative risk for hepatocellular carcinoma in sarcoidosis, was found to be 1.79. This is the first reported case of pancreatic adenocarcinoma associated with hepatic sarcoidosis. However, it is important to understand the difference between local sarcoid-like reaction versus systemic sarcoidosis [10]. A sarcoid-like reaction is a granuloma that usually occurs in the draining lymph nodes of cancer. This is most likely a local T-cell-mediated immune response to the shedding of cancer antigen. On the other hand, systemic sarcoidosis involves multiple organs, and has an abnormal chest x-ray and elevated ACE levels [11]. Treatment with steroids for at least 6 months is recommended in patients with symptomatic hepatic sarcoidosis. According to a report, one-third of patients treated with steroids had a complete clinical response, one-third had a partial response, and one-third had no response [2]. The definitive treatment of symptomatic hepatic sarcoidosis is liver transplantation; however, recurrence of the disease is possible in the new organ [2]. Our patient responded well to steroids initially. His symptoms, caused by obstructive jaundice, were first due to hepatic sarcoidosis and the second time owing to his pancreatic adenocarcinoma. In conclusion, hepatic sarcoidosis is a common entity and often occurs without pulmonary involvement. It presents as obstructive jaundice and has been associated with increased risk of hepatobiliary cancers. Our case highlights that biliary obstruction secondary to malignancy should be considered as a differential diagnosis for jaundice and hepatitis in sarcoidosis patients.
